# Regulation of the MDM2‐p53 pathway by the ubiquitin ligase HERC2

**DOI:** 10.1002/1878-0261.12592

**Published:** 2019-11-15

**Authors:** Jesús García‐Cano, Susana Sánchez‐Tena, Joan Sala‐Gaston, Agnès Figueras, Francesc Viñals, Ramon Bartrons, Francesc Ventura, Jose Luis Rosa

**Affiliations:** ^1^ Departament de Ciències Fisiològiques Institut d’Investigació de Bellvitge (IDIBELL) Universitat de Barcelona: Pavelló de Govern Spain

**Keywords:** DNA damage, HERC2, MDM2, NEURL4, p53, tumor suppressor

## Abstract

The p53 tumor suppressor protein is a transcription factor that plays a prominent role in protecting cells from malignant transformation. Protein levels of p53 and its transcriptional activity are tightly regulated by the ubiquitin E3 ligase MDM2, the gene expression of which is transcriptionally regulated by p53 in a negative feedback loop. The p53 protein is transcriptionally active as a tetramer, and this oligomerization state is modulated by a complex formed by NEURL4 and the ubiquitin E3 ligase HERC2. Here, we report that MDM2 forms a complex with oligomeric p53, HERC2, and NEURL4. HERC2 knockdown results in a decline in MDM2 protein levels without affecting its protein stability, as it reduces its mRNA expression by inhibition of its promoter activation. DNA damage induced by bleomycin dissociates MDM2 from the p53/HERC2/NEURL4 complex and increases the phosphorylation and acetylation of oligomeric p53 bound to HERC2 and NEURL4. Moreover, the *MDM2* promoter, which contains p53‐response elements, competes with HERC2 for binding of oligomeric, phosphorylated and acetylated p53. We integrate these findings in a model showing the pivotal role of HERC2 in p53‐MDM2 loop regulation. Altogether, these new insights in p53 pathway regulation are of great interest in cancer and may provide new therapeutic targets.

## Graphical abstract

The ubiquitin ligase MDM2 regulates protein levels of the p53 tumor suppressor protein. p53 is a transcription factor that regulates *MDM2* expression. This study reports the formation of a NEURL4/HERC2/oligomeric p53/MDM2 complex and how it participates in the stability of p53 and MDM2 proteins and in the transcriptional activation of p53 in response to DNA damage.

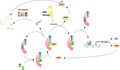

**Abbreviations**
ATM: ataxia/telangiectasia‐mutated
ATR: ataxia, telangiectasia and Rad3‐related
Bleo: bleomycin
BRCA1: breast cancer 1
CDDP: cis‐diamminedichloro platinum (II)
CHC: clathrin heavy chain
CHX: cycloheximide
CPH: cullin 7, Parc, HERC2
DNA‐PK: DNA‐dependent protein kinase
FBXL5: F‐box and leucine‐rich repeat protein 5
GAPDH: glyceraldehyde‐3‐phosphate dehydrogenase
HEK: human embryonic kidney
HERC2: HECT (homologous to the E6AP carboxyl terminus) and RCC1 (regulator of chromosome condensation 1) 2
IP: immunoprecipitation
MDM2: mouse double minute
mut: mutant
NEURL4: neuralized E3 ubiquitin protein ligase 4
NSCLC: non‐small‐cell lung cancer
NT: nontargeting
PAGE: polyacrylamide gel electrophoresis
PI: pre‐immune serum
PMSF: phenylmethylsulfonyl fluoride
PVDF: polyvinylidene fluoride
RE: response element
RING: really interesting new gene
shRNA: short hairpin RNA
siRNA: small interfering RNA
TP53: tumoral protein p53
wt: wild‐type
XPA: xeroderma pigmentosum antigen A

## 1 Introduction

The *TP53* gene encodes the p53 tumor suppressor protein which is a master transcription regulator of an extensive number of genes involved in apoptosis, proliferation, senescence, and metabolism among other cellular processes. In response to a wide range of cellular stresses including DNA damage, p53 activates this complex antiproliferative transcriptional program. *TP53* is the most frequently mutated gene in human cancer. Inactivating mutations of this gene are common, being linked to poor patient prognosis. Consistent with a tumor suppressor function, the *TP53* gene is mutated in more than half of all sporadic cancers and patients with Li‐Fraumeni syndrome (who are cancer prone) harbor germline *TP53* mutations (Kastenhuber and Lowe, 2017).

In nonstressed cells, p53 protein levels are low due to its proteasomal degradation after polyubiquitylation mediated mainly by the ubiquitin E3 ligase MDM2 (Haupt *et al.*, 1997; Kubbutat *et al.*, 1997; Michael and Oren, 2003). MDM2 also controls its own degradation through a self‐catalytic mechanism (Fang *et al.*, 2000; Honda and Yasuda, 2000). In stressed cells, MDM2 proteasomal degradation is stimulated and p53 becomes more stable and is activated (Horn and Vousden, 2007). During this activation process, p53 oligomerizes and is phosphorylated by kinases at several threonine/serine residues and acetylated by acetyltransferases at multiple lysine residues (Cubillos‐Rojas *et al.*, 2014; Itahana *et al.*, 2009; Tang *et al.*, 2008). Activated p53 binds to p53 response elements located in the promoter of its target genes to activate or repress their transcription (Fischer *et al.*, 2015). *MDM2* is one of these p53 target genes. Hence, this forms a negative feedback loop (Karni‐Schmidt *et al.*, 2016; Manfredi, 2010).

The p53 protein contains a functional domain at the C terminus of its structure that permits its oligomerization. It is believed that in nonstressed cells, p53 exists predominantly in a dimer state. Upon a stress signal, p53 concentration increases, shifting to a tetramer state that binds with more affinity to DNA and regulating the transcription of its target genes (Kawaguchi *et al.*, 2006; Stommel *et al.*, 1999; Weinberg *et al.*, 2004). The oligomerization state also affects other aspects of p53 function such as its post‐translational modifications, its degradation, and its interaction with other proteins (Chène, 2001; Kamada *et al.*, 2016). Since acetylation is indispensable for p53 activation (Tang *et al.*, 2008) and p53 oligomerization is essential for its acetylation (Itahana *et al.*, 2009), p53 oligomerization is a critical step during its transcriptional activation. Most mutations in the oligomerization domain of p53 prevent its oligomerization, its binding to DNA, its transcriptional activity and are associated with tumor progression as occurs in patients with Li‐Fraumeni and Li‐Fraumeni‐like syndromes (Davison *et al.*, 1998; Lomax *et al.*, 1998). The ubiquitin E3 ligase HERC2 and the NEURL4 protein are required for oligomerization and subsequent transcriptional activation of p53 (Cubillos‐Rojas *et al.*, 2014, 2017).

HERC2 belongs to the large HERC family of ubiquitin E3 ligases. Members of this family contain more than one regulator of chromosome condensation 1 (RCC1)‐like domain (RLD) and a homologous to the E6AP carboxyl terminus (HECT) ubiquitin ligase domain (García‐Cano *et al.*, 2019; Sánchez‐Tena *et al.*, 2016). HERC2 is the only member of this small family that contains a p53‐binding domain named CPH due to its presence in cullin 7 (CUL7), Parkin‐like cytoplasmic (PARC), and HERC2 itself. Proteins with a CPH domain bind p53 and regulate its activity in different ways. Thus, whereas CUL7 and PARC promote cell growth by antagonizing p53 functions, HERC2 activates p53, thus inhibiting cell cycle progression (Andrews *et al.*, 2006; Cubillos‐Rojas *et al.*, 2014; Kasper *et al.*, 2006; Kaustov *et al.*, 2007; Nikolaev *et al.*, 2003). Substrates of HERC2, such as XPA, BRCA1, USP33, and FBXL5, relate this E3 ligase to cellular processes such as DNA repair, centrosome structure, and iron metabolism (García‐Cano *et al.*, 2019; Sánchez‐Tena *et al.*, 2016). Genetic studies have associated the *HERC2* locus with human pigmentation, neuronal disorders, and cancer (for review, see Refs García‐Cano *et al.*, 2019; Sánchez‐Tena *et al.*, 2016). For example, a neurodevelopmental delay featuring Angelman syndrome and autism spectrum disorder has been attributed to a homozygous missense mutation [NM_004667.5:c.1781C>T (p.Pro594Leu)] in the *HERC2* gene (Harlalka *et al.*, 2013; Puffenberger *et al.*, 2012) or to a homozygous 286‐kb deletion between the contiguous genes *HERC2* and *OCA2* (chr15: g. 28143765_28429460 del) (Morice‐Picard *et al.*, 2016). Mutations in *HERC2* have also been described in leukemia (Johansson *et al.*, 2018), and gastric and colorectal carcinomas [NM_004667.5:c.541delA (p.Ser181ValfsX85)] (Yoo *et al.*, 2011).

The molecular mechanism involved in the regulation of the transcriptional activity of p53 is complex. Although numerous studies have contributed to an emerging model of regulation, it is necessary to understand how newly identified interactors integrate with this model. The formation of a complex between HERC2 and p53 led us to investigate whether HERC2 participates in the negative feedback loop of p53 and MDM2, whereby p53 activates the transcription of MDM2, and MDM2 inhibits the transcriptional activity of p53, facilitates its cytoplasmic localization, and promotes its degradation. In the current study, we identified an interaction between HERC2, p53, and MDM2 in which p53 oligomerization is essential for the formation of this complex. HERC2 regulates *MDM2* gene expression by a p53‐dependent transcriptional mechanism. Moreover, the HERC2‐p53‐MDM2 interaction is regulated by DNA damage. Following DNA damage caused by bleomycin, oligomeric p53 is phosphorylated and acetylated, and MDM2 is dissociated from the complex. Our findings also show that the *MDM2* promoter containing p53 response elements, binds acetylated, phosphorylated, and oligomeric p53, displacing it from the complex with HERC2. These data have significant implications in the model of regulation of p53 activity, revealing that HERC2 is a critical component in regulation of the p53‐MDM2 loop.

## 2 Methods

### 2.1 Cell lines, culture conditions, and treatments

U2OS, HEK293T, A549, and H1299 cell lines were obtained from ATCC (Manassas, VA, USA) and cultured in Dulbecco’s modified Eagle’s medium (01‐055‐1A) supplemented with 10% fetal bovine serum (04‐007‐1A), 100 U·mL^−1^ penicillin, and 0.1 mg·mL^−1^ streptomycin sulfate (03‐031‐1B) and 2 mm l‐glutamine (03‐020‐1B) from Biological Industries (Beit HaEmek, Israel). Cells were treated where indicated with 20 μg·mL^−1^ cycloheximide (C7698), 10 μg·mL^−1^ (0.015 U·mL^−1^) bleomycin sulfate (B5507) or 10 μm MG132 (C2211) from Sigma‐Aldrich/Merck (Darmstadt, Germany).

### 2.2 Plasmids and siRNAs transfection

pcDNA3‐Flag‐MDM2 plasmid, p53 constructs (wt, R337C, L344P, NLS, NES, and p53‐CFP) and Myc‐tagged F3 fragment from HERC2 (residues 2292–2923) containing the CPH domain were obtained from Burgering (Brenkman *et al.*, 2008), Zhang (Itahana *et al.*, 2009), and Ohta (Wu *et al.*, 2010), respectively. For gene interference, custom double‐stranded siRNA oligonucleotides were obtained from GeneCust (Boynes, France) and previously tested among others elsewhere (Cubillos‐Rojas *et al.*, 2014). Forward sequences were nontargeting (NT): 5′‐UAGCGACUAAACACAUCAAdTdT‐3′, HERC2: 5′‐ACUGUAGCCAGAUUGAAAdTdT‐3′, and MDM2: 5′‐GAAGUUAUUAAAGUCUGUUdTdT‐3′, along with their respective reverse oligonucleotides. Interference with siRNAs was carried out by transfecting the oligonucleotides using the calcium phosphate method as described elsewhere (Cubillos‐Rojas *et al.*, 2014). Plasmid transfection was performed using the Lipofectamine LTX method (15338; Invitrogen, Carlsbad, CA, USA) according to the manufacturer’s instructions.

### 2.3 Protein extraction, PAGE, western blot, and antibodies

For protein extraction, cells were washed twice in ice‐cold PBS after media were discarded and lysed by scraping with 100 μL lysis buffer every one million cells (lysis buffer was composed by 0.3% CHAPS with 100 mm NaCl in 10 mm Tris/HCl, pH = 7.5; supplemented with protease and phosphatase inhibitors: 50 mm NaF, 50 mm β‐glycerophosphate, 1 mm PMSF, 1 mg·mL^−1^ benzamidine, 5 μg·mL^−1^ leupeptin, 1 μg·mL^−1^ pepstatin A, 5 μg·mL^−1^ aprotinin, 1 μm E64, and 1 mm Na_3_VO_4_). Lysates were centrifuged at 13 000 ***g*** for 10 min at 4 °C, and pellets were discarded. Protein concentrations were quantified using a BCA kit (23223 and 23224) supplied by Thermo Scientific (Waltham, MA, USA) according to the manufacturer’s instructions. Gradient (3–15%) polyacrylamide gel electrophoresis and protein transfer were performed as previously described (Cubillos‐Rojas *et al.*, 2010). Band intensity was measured, when indicated, using imagej software (Collins, 2007; Schneider *et al.*, 2012).

Antibodies were from the following companies: BD Transduction (HERC2, 612366, Franklin Lakes, NJ, USA), Abcam (MDM2 2A10, ab16895, Cambridge, UK), Santa Cruz Biotechnology (Dallas, TX, USA) [p53 DO‐1 (mouse), sc‐126; p53 N‐19 (goat, used in western blots for immunoprecipitation assays), sc‐1314; α‐tubulin, sc‐53646; p21, sc‐397 and NEURL4, sc‐243602], Sigma‐Aldrich/Merck (Flag M2, F1804), Roche (c‐Myc, 11667149001, Basel, Switzerland), and Cell Signaling Technology (P‐p53 S15, #9284 and Ac‐p53 K382, #2525, Danvers, MA, USA).

### 2.4 Immunoprecipitation, oligo pulldown, and oligomerization assays

For immunoprecipitation, 1 mg protein from cell lysates was incubated at 4 °C for 2 h on a rotatory wheel with polyclonal HERC2 antibody bvg3 (generated against residues 1–199 as described elsewhere, Cubillos‐Rojas *et al.*, 2014), Myc antibody (11667149001) from Roche, or anti‐Flag M2 Affinity Gel (A2220) from Sigma/Merck. For the HERC2 and Myc immunoprecipitations, protein A‐conjugated sepharose slurry (71‐7090‐00) from GE Healthcare (Chicago, IL, USA) was washed twice in wash buffer (lysis buffer described in Section 2.3, without inhibitors) and added to the lysates, which were left for incubation in the same conditions for one additional hour. After this time, lysates were centrifuged at 2500 ***g*** for 2 min at 4 °C and washed four times in 1 mL wash buffer. Pellets were resuspended in 2× loading buffer [0.5 m Tris/HCl, pH = 8.5; 40 mg·mL^−1^ LDS, 0.3 mg·mL^−1^ EDTA, 20% glycerol, 0.0375% Coomassie blue, 0.0125% phenol red, and 100 mm dithiothreitol (DTT)] and stored at −20 °C until they were analyzed. Inputs represent 1/25 from total cell lysates.

Protein lysates for oligo pulldown were harvested by scraping in oligo pulldown lysis buffer [100 mm KCl, 10 mm HEPES pH = 7.9, 10% glycerol, 1 mm DTT, 5 mm MgCl_2_, 0.1% Nonidet P‐40 (NP‐40) substitute (786–511) from GBiosciences (St. Louis, MO, USA), supplemented with the protease and phosphatase inhibitors mentioned in Section 2.3] and centrifuged at 13 000 ***g*** for 10 min at 4 °C. Pellets were discarded and supernatants were incubated overnight at 4 °C on a rotatory wheel with 1 μg double‐stranded 5′‐biotinylated oligonucleotides along with 1 μg poly‐dIdC (sc‐286691A) from Santa Cruz Biotechnology. The oligonucleotide forward sequences were as follows: Sp1 (murine *Col1a1* promoter, as negative control): 5′‐BIO·GGAACAGAAGGGGAGGAGC‐3′; p21: 5′‐BIO·GTCAGGAACATGTCCCAACATGTTGAGCTC‐3′; MDM2wt: 5′‐BIO·GAGCTGGTCAAGTTCAGACACGTTCCGAAA‐3′ and MDM2mut: 5′‐BIO·GAGCTGGTTAAATTCAGATACATTCCGAAA‐3′, along with their respective unmodified reverse oligonucleotides. Streptavidin‐conjugated agarose slurry (17‐5113‐01) from GE Healthcare was washed twice in wash buffer (oligo pulldown lysis buffer with no NP‐40 substitute or inhibitors) and added to the lysates, which were left for incubation in the same conditions for one additional hour. After that, lysates were centrifuged at 1250 ***g*** and 4 °C for 1 min and washed four times in the same wash buffer. Pellets were resuspended in 2× loading buffer and stored at −20 °C until they were analyzed. Inputs represent 1/25 from total cell lysates.

For the oligomerization assays, glutaraldehyde solution was added to the pulldown products to a final concentration of 0.04% in wash buffer and incubated for 30 min on ice with mild rocking before loading buffer was added (Cubillos‐Rojas *et al.*, 2014).

### 2.5 Quantitative real‐time PCR

Total RNA isolation, reverse transcription, and quantification were performed as previously described (Cubillos‐Rojas *et al.*, 2017). Taqman assay probes for *MDM2* (Hs00540450_s1) and *GAPDH* (Hs99999905_m1) were obtained from Thermo Scientific.

### 2.6 Luciferase assays

U2OS and H1299 cells were transfected with either pGL2‐hmdm‐Hx (*wt MDM2* promoter) or pGL2‐hmdm‐Px (Δ*RE1 MDM2* promoter) luciferase‐expressing plasmids given by Oren (Zauberman *et al.*, 1995), and a β‐galactosidase construct. Luciferase activity was quantified using a Luciferase Assay System (E1500) from Promega (Madison, WI, USA) according to the manufacturer’s instructions. Luciferase values were normalized using β‐galactosidase activity measured using the Luminescent β‐Galactosidase Detection Kit II (631712) from Clontech/Takara (Kusatsu, Japan). Luminescence levels are expressed as fold induction versus the nontargeting siRNA‐transfected controls.

### 2.7 p53 competition experiments

For p53 competition, cells were treated with bleomycin for 3 h, lysed in oligo pulldown lysis buffer as described in Section 2.4, and extracts were incubated overnight with either pGL2‐hmdm‐Hx plasmid (Section 2.6) or with minimum promoter‐containing pGL2 basic. Lysates were immunoprecipitated as indicated earlier in Section 2.4.

### 2.8 Lentivirus production and shRNA gene interference

For effective gene interference, lentiviral vectors were produced in HEK293T. Cells were transfected with 7 μg pMD2.G, 7 μg psPAX2 (VSV‐G), and 7 μg of either empty pLKO.1 puro or pLKO.1‐shHERC2 (SHCLNG‐NM_004667; Sigma‐Aldrich/Merck) using the calcium phosphate method as described elsewhere (Cubillos‐Rojas *et al.*, 2014). Media were changed the day after. Twenty‐four hours later, media (which contained the lentiviral particles produced) were collected, filtered using Millex‐HV 0.45 μm PVDF filters (SLHV033RB; Millipore, Burlington, MA, USA), and stored at 4 °C. Fresh media was added to the cells. The same procedure was performed the day after. Both media collections containing lentiviral vectors were merged and stored in aliquots at −80 °C. Host A549 and H1299 cells were seeded at a confluence of 40–50% in 6‐well plates. The day after, 300 µL lentivirus‐containing media were added to each well and made up to a total volume of 1 mL/well with fresh medium supplemented with polybrene (H9268; Sigma‐Aldrich/Merck) at a final concentration of 5 µg·mL^−1^. Media were changed the day after. After 24 h, puromycin was added at a final concentration of 1.5 µg·mL^−1^ for A549 and 3 µg·mL^−1^ for H1299 and left for at least 72 h before experiments were performed. Noninfected cells were used as a selection control. Cells were routinely maintained in puromycin‐containing media, which was removed prior to each experiment.

### 2.9 Cell growth and clonogenic assays

For viability assays, the indicated cell lines were seeded to a final concentration of 2 × 10^4^ cells/well in three wells of a 24‐well plate per condition and time point. Every 24 h, 1/10 volumes of MTT (M5655; Sigma/Merck) (5 mg·mL^−1^ in PBS) was added per well to the media in one of the plates and incubated for 1 h at 37 °C in the cell incubator. Media were then discarded, and formazan crystals were recovered with DMSO and absorbance at λ = 570 nm was determined using a 96‐well plate spectrophotometer. The results are reported as percentage versus the 24‐h time point.

For cell growth assays with crystal violet staining, the indicated cell lines were seeded to a final concentration of 2 × 10^4^ cells/well in three wells of a 24‐well plate per condition and time point. Every 24 h, the media in one of the plates were discarded, cells were washed with 1X  PBS with mild rocking for 5 min at room temperature and incubated with 0.2% crystal violet (C0775; Sigma‐Aldrich/Merck) dissolved in 0.5% glutaraldehyde in water for 15 min with mild rocking at room temperature. The excess dye was washed with running tap water and allowed to dry overnight at room temperature upside down. Dye that had adhered to the cells was recovered with 10% acetic acid and absorbance at λ = 595 nm was determined using a 96‐well plate spectrophotometer. The results are reported as percentage versus the 24‐h time point.

Clonogenic assays were performed by seeding 2000 cells/well in 6‐well plates and dying them with crystal violet solution, as described above, 12 days after. The results are reported as a percentage versus cells infected with the lentivirus carrying the empty pLKO vector.

### 2.10 Cisplatin dose–response assays

The indicated cell lines were seeded at a final concentration of 2 × 10^4^ cells/well in a 24‐well plate and left overnight. A day later, the media were discarded and replaced with fresh media containing the indicated concentrations of cisplatin (CDDP) (P4394; Sigma‐Aldrich/Merck). After 48 h, cells were dyed with MTT as described in Section 2.9.

### 2.11 Statistical analysis

The results shown are the means of, at least, three independent experiments ± SEM. Significance was calculated by Student’s *t*‐test using prism 5.00 software from GraphPad (San Diego, CA, USA) and is indicated as follows: *P ≤ 0.05; **P ≤ 0.01; ***P ≤ 0.001.

## 3 Results

### 3.1 MDM2 binds HERC2 through oligomerized p53

**Figure 1:**
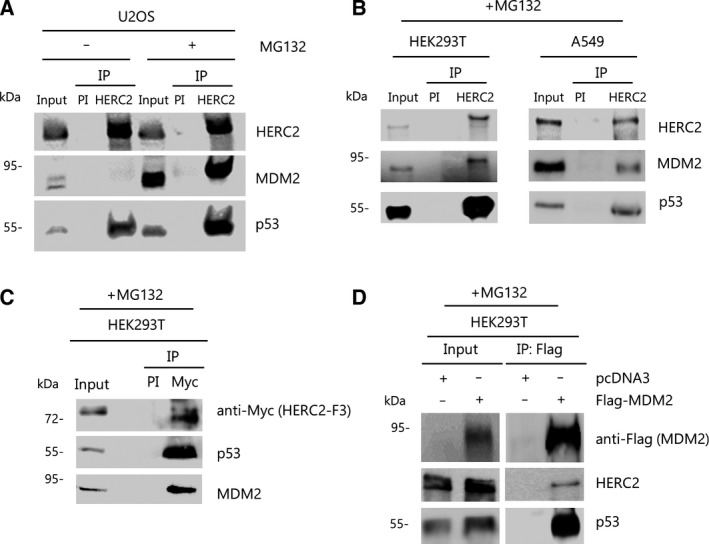
HERC2 binds MDM2 and p53. (A) U2OS cells were either untreated (−) or treated (+) with proteasome inhibitor MG132 for 6 h and protein extracts were immunoprecipitated (IP) against HERC2 using bvg3 antibody or incubated with pre‐immune serum (PI) as a negative control. Immunoprecipitation products were immunoblotted against the indicated proteins. (B) HEK293T and A549 cells were treated with MG132, and immunoprecipitation was carried out as in (A). (C) HEK293T cells were transfected with Myc‐tagged, CPH‐containing HERC2 F3 region for 24 h, and immunoprecipitation against Myc epitope was performed as described for (A) after 6 h of MG132 pretreatment. (D) HEK293T cells were transfected (+) with Flag‐MDM2 or empty pcDNA3‐Flag plasmid as a control for 24 h. Protein extracts were immunoprecipitated against Flag epitope. Immunoprecipitation products were processed as described in (A). Shown data are representative of, at least, three independent experiments.

**Figure 2:**
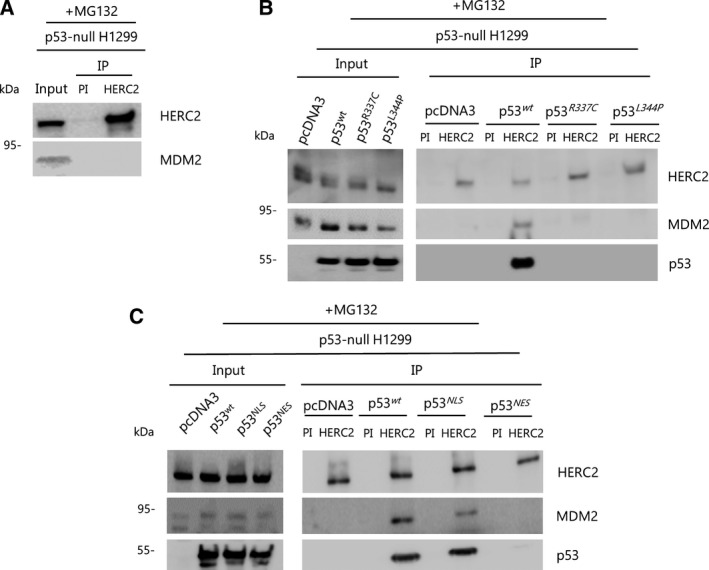
Oligomerized p53 is required for the binding of MDM2 to HERC2. (A) p53‐null H1299 cells were treated with MG132 for 6 h, and protein extracts were immunoprecipitated (IP) against HERC2 with bvg3 antibody or incubated with pre‐immune serum (PI) as a negative control. Immunoprecipitation products were immunoblotted against the indicated proteins. (B, C) H1299 cells were transfected with the indicated p53 constructs (for more details about constructs, see Itahana et al., 2009) for 24 h. Cells were treated for the last 6 h with MG132, and immunoprecipitation was carried out as described in (A). Shown data are representative of, at least, three independent experiments.

As it has been reported that HERC2 binds p53 (Cubillos‐Rojas *et al.*, 2014, 2017), and given that MDM2 is a well‐known interactor with p53 (Moll and Petrenko, 2004; Wu *et al.*, 1993), we decided to investigate whether these two events occur simultaneously. A simultaneous interaction of HERC2 with both endogenous p53 and MDM2 can be observed in immunoprecipitation experiments using specific anti‐HERC2 (bvg3) antibody in U2OS cells (Fig. 1A). The interaction of HERC2 with MDM2 is scarce, probably due to the low levels of MDM2, which makes it difficult to detect in protein complexes. It is well known that the inhibition of proteasome activity increases MDM2 and p53 levels. Thus, we decided to analyze this interaction upon proteasome inhibition by previous treatment with MG132 for 6 h. Under these conditions, MDM2 levels increased and a strong interaction was detected (Fig. 1A). This finding was also observed in other cell lines such as HEK293T and A549 upon proteasome inhibition as well (Fig. 1B). One of the defining protein domains of HERC2 is CPH, which is also present in Cul7 and PARC and is known to bind to p53 (Andrews *et al.*, 2006; Cubillos‐Rojas *et al.*, 2014; Kasper *et al.*, 2006; Kaustov *et al.*, 2007; Nikolaev *et al.*, 2003). In the presence of MG132, we observed binding of p53 as well as MDM2 to the Myc‐tagged, CPH domain‐containing F3 region (residues 2292–2923) of HERC2 (Fig. 1C). Reciprocally, HERC2 and p53 also co‐immunoprecipitated with Flag‐MDM2 in transfected HEK293T cells (Fig. 1D). In order to assess whether the interaction between MDM2 and HERC2 was p53‐dependent, we performed the same immunoprecipitation approach in p53‐null H1299 cells. After pretreating cells with MG132, MDM2 does not bind HERC2 in the absence of p53, as shown in Fig. 2A. We wanted to know whether the oligomerization of p53 was necessary for MDM2 binding to the HERC2‐p53 complex. We therefore transfected H1299 cells with either wild‐type (*wt*) p53 or with the *R337C* and *L334P* p53 mutant variants found in Li‐Fraumeni syndrome, which are unable to oligomerize (Davison *et al.*, 1998; Itahana *et al.*, 2009; Lomax *et al.*, 1998). Only in the *wt* p53‐transfected H1299, it was possible to coimmunoprecipitate p53 and MDM2 with HERC2 (Fig. 2B). Moreover, we tested p53 mutants for nuclear localization signal (*NLS*) and for nuclear export sequence (*NES*) since the latter is also known to be defective in oligomerization (Itahana *et al.*, 2009). Immunoprecipitation of endogenous HERC2 yielded evidence for MDM2 binding to p53 along with HERC2 only in H1299 cells expressing the *wt*‐ or *NLS*‐p53. In contrast, immunoprecipitation of HERC2 in the presence of the *NES* mutant failed to retrieve either p53 or MDM2 (Fig. 2C). Altogether, these results show that MDM2 binds HERC2 through oligomerized p53.

### 3.2 HERC2 regulates MDM2 expression through a p53‐dependent transcriptional mechanism

**Figure 3:**
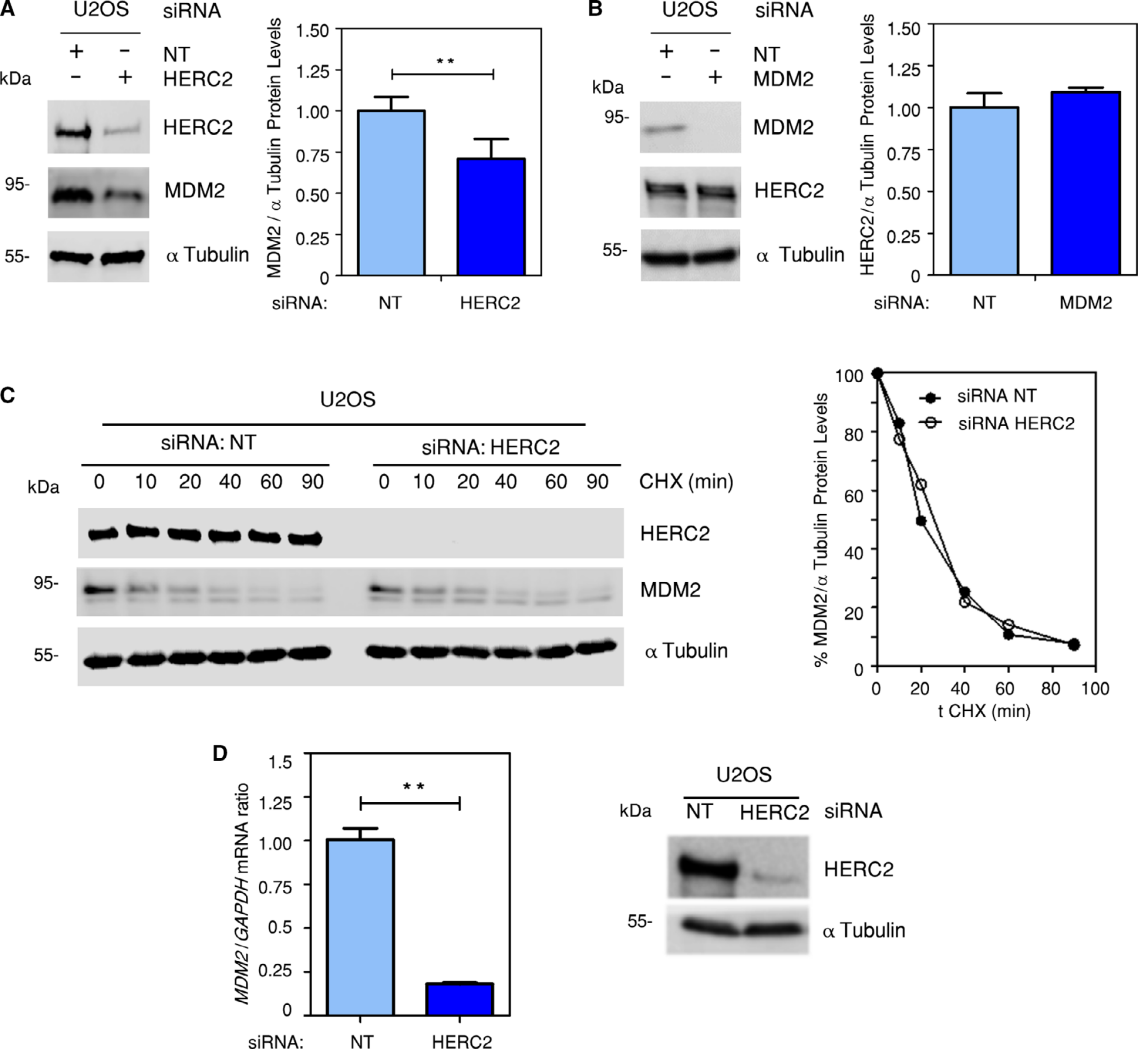
HERC2 regulates MDM2 expression. (A) Nontargeting (NT) or HERC2‐directed siRNAs were transfected (+) into U2OS for 72 h. Protein extracts were immunoblotted against the indicated proteins. α‐Tubulin was used as a loading control. The chart indicates band quantification of three independent experiments. (B) Nontargeting (NT) or MDM2‐directed siRNAs were transfected (+) into U2OS for 72 h. Protein extracts were immunoblotted against the indicated proteins. α‐Tubulin was used as a loading control. The chart indicates band quantification of three independent experiments. (C) Nontargeting (NT) or HERC2‐directed siRNAs were transfected into U2OS for 72 h. Cells were treated, during the last 90 min, with 20 µm of the protein‐synthesis inhibitor cycloheximide (CHX) for the indicated times. Protein extracts were immunoblotted against the indicated proteins. α‐Tubulin was used as a loading control. The chart indicates band quantification. (D) Nontargeting (NT) or HERC2‐targeted siRNAs were transfected into U2OS for 72 h. qRT–PCR experiments were performed with total cDNA using specific Taqman probes against *MDM2* and *GAPDH* as a loading control. Parallel protein extracts were immunoblotted against the indicated proteins. α‐Tubulin was used as a loading control. Data were analyzed by Student’s *t*‐test. Error bars indicate SEM from three independent experiments.

Due to their ubiquitin ligase activity, binding of HERC2 to MDM2 could suggest a possible mechanism of ubiquitylation and subsequent proteasome‐dependent degradation of one of these two proteins. To figure that out, we knocked HERC2 down by transfecting U2OS cells with specific siRNA. HERC2 knockdown yielded a reduction in MDM2 protein levels (Fig. 3A) compared to those in nontargeting siRNA‐transfected cells. To fully rule out similar reciprocal activity, we performed knockdown of MDM2 by siRNA transfection and evaluated HERC2 expression also in U2OS cells. In this case, HERC2 protein levels were not affected by MDM2 downregulation (Fig. 3B). We then decided to investigate the mechanism by which MDM2 levels diminish upon HERC2 knockdown. We performed a time‐course experiment in the presence of protein translation inhibitor cycloheximide (Kao *et al.*, 2015; Wettstein *et al.*, 1964) after transfecting either HERC2‐directed or nontargeting siRNAs in U2OS cells. As indicated in Fig. 3C, no significant differences in protein stability patterns were observed following HERC2 knockdown. However, *MDM2* mRNA levels were effectively reduced upon HERC2 depletion compared to the nontargeting control siRNA transfection (Fig. 3D). These data indicate that the decline observed in MDM2 protein levels upon HERC2 knockdown relies on a reduction in its transcription rates rather than on a mechanism affecting protein stability.

**Figure 4:**
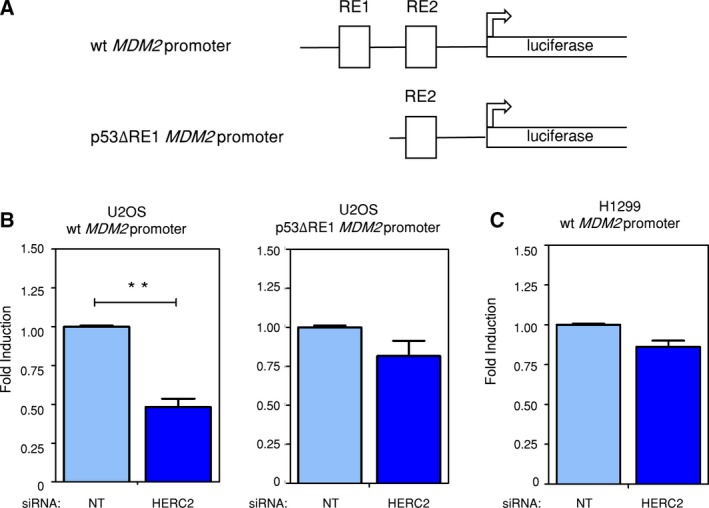
HERC2 knockdown reduces *MDM2* promoter activation in a wt p53 context. (A) Schematic diagram of the *MDM2* promoter region in pGL2‐hmdm‐Hx (wt *MDM2* promoter) and pGL2‐hmdm‐Px (p53ΔRE1 *MDM2* promoter) plasmids (for more details see Zauberman et al., 1995). (B, C) U2OS or p53‐null H1299 cells were transfected with either nontargeting (NT) or HERC2‐directed siRNAs for 72 h. During the last 24 h, cells were transfected with luciferase‐expressing vectors under the control of either wt *MDM2* promoter or p53ΔRE1 *MDM2* promoter where indicated and luciferase activity was measured. The charts represent normalized luciferase measurements versus the NT series. Data were analyzed by Student’s *t*‐test. Error bars indicate SEM from three independent experiments.

The *MDM2* gene is under the control of a promoter‐containing two p53 response elements (RE1 and RE2) (Wu *et al.*, 1993). To confirm the involvement of HERC2 in MDM2 transcriptional regulation through p53, plasmids containing luciferase gene under the control of either *wt* (pGL2‐hmdm‐Hx) or p53 response element 1‐lacking (Δ*RE1*) (pGL2‐hmdm‐Px) *MDM2* promoter (Fig. 4A) were transfected into U2OS cells. HERC2 knockdown significantly reduces luciferase activity in cells transfected with the *wt* promoter‐carrying plasmid but not with the *ΔRE1* promoter (Fig. 4B). In addition to this, HERC2 knockdown did not affect luciferase activity on the *wt* promoter in p53‐null H1299 cells (Fig. 4C). Together, these results suggest that HERC2 controls MDM2 levels through a p53‐dependent transcriptional mechanism.

### 3.3 Binding of p53, but not HERC2 nor MDM2, to MDM2 promoter

**Figure 5:**
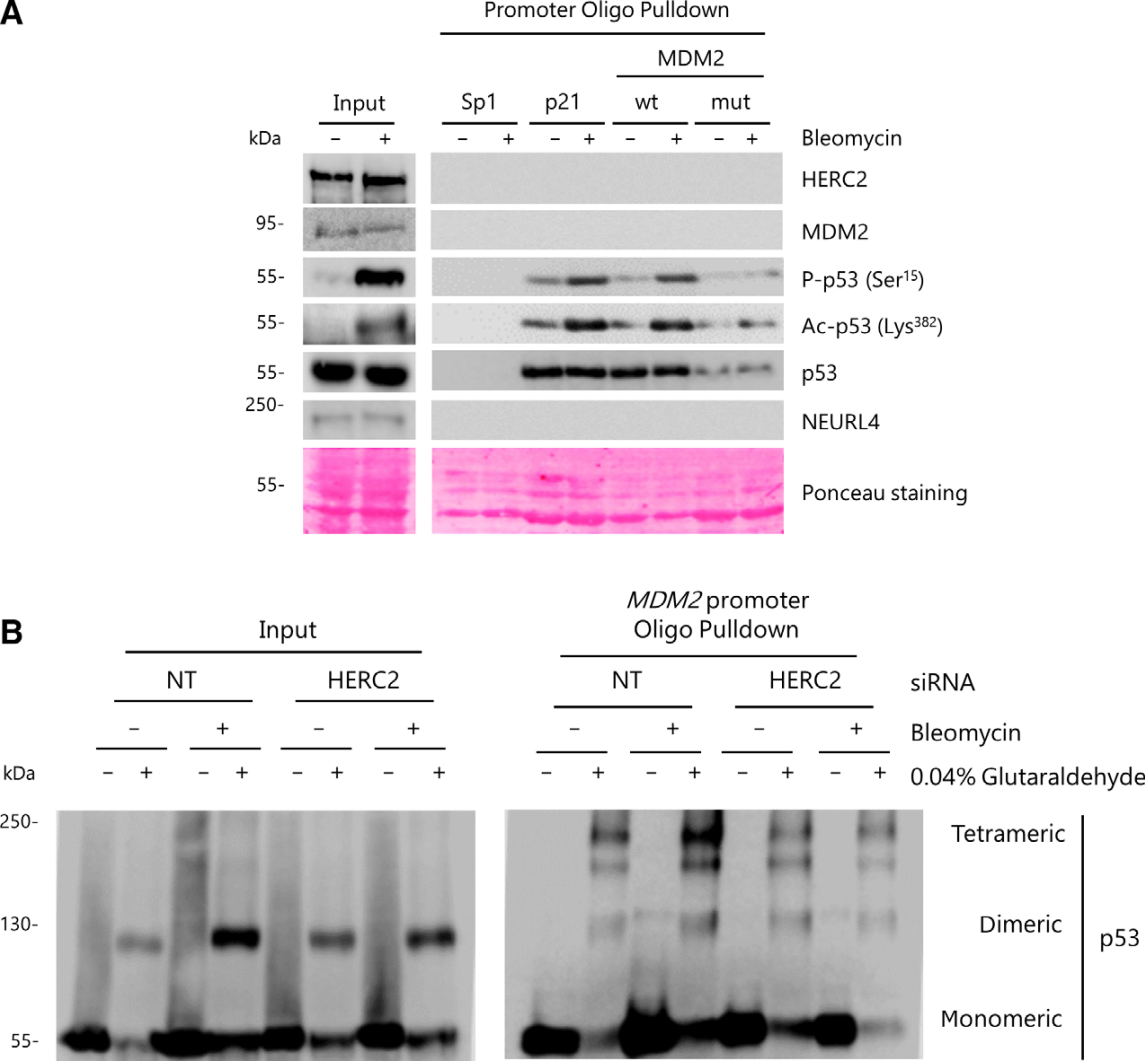
p53, but not HERC2 nor MDM2, binds biotinylated oligonucleotides from promoter regions. (A) U2OS cells were treated with bleomycin for 3 h (+) or untreated (−) as a control, and oligo pulldown experiments were performed with protein extracts. Pulldown products were immunoblotted against the indicated proteins. Shown data are representative of three independent experiments. The membrane from the 55‐kDa band was stained with Ponceau S solution (B) U2OS cells were transfected with either nontargeting (NT) or HERC2‐directed siRNAs for 72 h. During the last 3 h, cells were either treated with bleomycin (+) or untreated as a control (−) and oligo pulldown experiments were performed as in (A). Pulldown products were split into two aliquots. One of them was incubated with 0.04% glutaraldehyde before adding loading buffer and the other one remained untreated as a control. Pulldown products in both cases were immunoblotted against p53. Shown data are representative of two independent experiments.

Since *MDM2* expression is activated by p53, especially under DNA‐damaging stress conditions (Toledo and Wahl, 2006), we wondered whether the HERC2‐p53‐MDM2 complex described above is present in the *MDM2* promoter region. To this end, we performed oligo pulldown experiments in U2OS cells with either p53 RE1‐*wt* (wt) or p53 RE1‐mutant (*mut*) *MDM2* promoter biotinylated oligonucleotides. The p53 responsive element (RE) from the *p21* promoter was used as a positive control due to its affinity for p53 and murine *Col1a1* promoter Sp1 sequence was used as a negative control. Cells were either treated with bleomycin, a DNA‐damaging agent known to promote p53 activation (Cubillos‐Rojas *et al.*, 2014; Panchanathan *et al.*, 2015 and Fig. S1), or untreated as a control. As expected, the results show that p53 binds to *p21* promoter, with an increase of phosphorylated and acetylated form upon bleomycin treatment (Fig. 5A). Binding of p53, both total and activated, to the *wt MDM2* promoter was similar. However, this binding was drastically lower with the *mut MDM2* promoter. No HERC2 nor MDM2 binding was detectable in either conditions. NEURL4, a regulator of p53 transcriptional activity through interaction with HERC2 and p53 (Cubillos‐Rojas *et al.*, 2017), was also analyzed and similar results were obtained. These data show that HERC2, NEURL4, and MDM2 do not bind the promoter regions where p53 is bound. Since p53 transcriptional activity requires its tetramerization (Itahana *et al.*, 2009), we wanted to assess the oligomerization state of p53 on the *MDM2* promoter. To this end, we carried out oligo pulldown experiments in bleomycin‐treated or bleomycin‐untreated U2OS cells. Protein samples were then processed either in the presence or in the absence of 0.04% glutaraldehyde solution as a crosslinker to visualize oligomerization. We observed a high relative amount of monomeric and dimeric p53 in total protein extracts (Input) whereas tetrameric p53 was undetectable (Fig. 5B, left panel). The dimeric/monomeric ratio notably rose upon bleomycin treatment. This increase was reduced by *HERC2* interference through siRNA transfection (Fig. 5B, left panel). The tetrameric form was enriched in the protein extracts bound to the biotinylated *p53 RE1* from the *MDM2 wt* promoter (oligo pulldown). *HERC2* silencing led to a lower amount of tetrameric p53 bound to the *MDM2* promoter both in basal and in bleomycin‐treated conditions (Fig. 5B, right panel). These findings show the absence of HERC2, MDM2, or NEURL4 proteins on the *MDM2* promoter, the specific binding of p53 to *MDM2* promoter and the increase of oligomerized/activated p53 bound to this promoter after DNA damage caused by bleomycin.

### 3.4 Regulation of HERC2‐p53‐MDM2 complex formation

**Figure 6:**
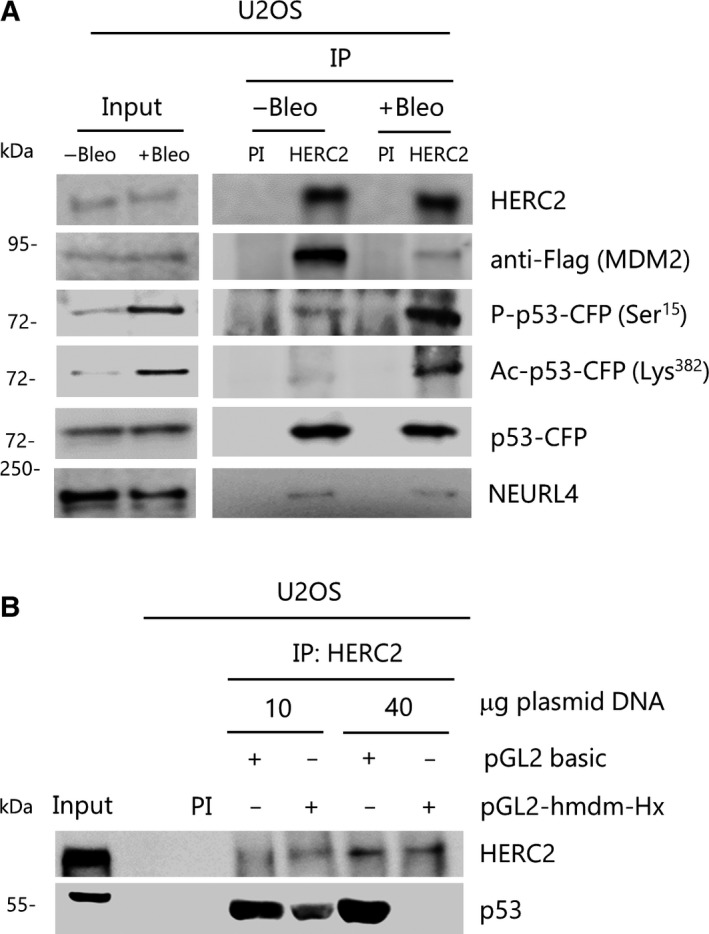
Regulation of HERC2‐p53‐MDM2 complex formation. (A) U2OS cells were transfected with Flag‐MDM2 and p53‐CFP for 24 h. During the last 3 h, cells were either treated with bleomycin (+Bleo) or remained untreated as a control (−Bleo) and protein extracts were immunoprecipitated (IP) with either bvg3 antibody against HERC2 or with pre‐immune serum (PI) as a negative control. Immunoprecipitation products were immunoblotted against the indicated proteins. (B) U2OS cells were treated with bleomycin for 3 h. Protein extracts were incubated overnight (+) with the indicated amounts of either *MDM2* promoter‐containing luciferase‐expressing plasmid (pGL2‐hmdm‐Hx) or with minimum promoter‐containing luciferase‐expressing plasmid (pGL2 basic) as a control and immunoprecipitated as in (A). Shown data are representative of, at least, two independent experiments.

During activation of p53‐regulated gene transcription such as that of *p21* or *MDM2* genes, MDM2 and p53 proteins should be released from the HERC2‐p53‐MDM2 complex according to the results described above. As shown earlier, p53 bound to the promoters of these genes upon activation/DNA damage is preferentially oligomerized, acetylated and phosphorylated. To analyze this further, we performed immunoprecipitation of HERC2 in U2OS cells both in basal and in bleomycin‐treated conditions. To detect MDM2, cells were transfected with Flag‐MDM2 construct. Since antibodies against phosphorylated p53, acetylated p53, and the antibody against HERC2 used to immunoprecipitate (bvg3) were produced in rabbits, cells were also transfected with p53‐CFP construct to avoid interference of the antibodies against active p53 with the immunoglobulins from rabbit sera (immunoglobulin heavy‐chain molar mass is about 50 kDa). Bleomycin treatment induced both phosphorylation and acetylation of p53 (Fig. 6A, Input). We observed that MDM2 binding to the HERC2‐p53 complex was greatly reduced after bleomycin treatment whereas p53 binding was not notably affected. In these conditions, phosphorylated and acetylated p53 remains bound to HERC2. NEURL4 is also present in the complex (Fig. 6A). Since there were no significant differences in binding between HERC2 and p53 after bleomycin treatment, these results suggest that only a small fraction of p53 is bound to promoters under these conditions. We hypothesized that promoters containing p53 response elements compete with HERC2 for the binding of p53. To test this hypothesis, we performed a competition experiment in which plasmids containing the *wt MDM2* promoter (pGL2‐hmdm‐Hx) (Fig. 4A) were incubated with bleomycin‐treated U2OS cell lysates overnight prior to immunoprecipitation. The same luciferase‐expressing plasmid backbone with minimum promoter (no *MDM2* promoter sequences) pGL2 basic was used as a negative control. Pre‐incubation of the pGL2‐hmdm‐Hx with the protein extracts effectively abolished p53 binding to HERC2 in a dose‐dependent manner. These data suggest that p53 detaches from HERC2 and binds target gene promoter (Fig. 6B).

### 3.5 Stable HERC2 interference enhances cell growth and desensitizes cells against cisplatin in the presence of wt p53

**Figure 7:**
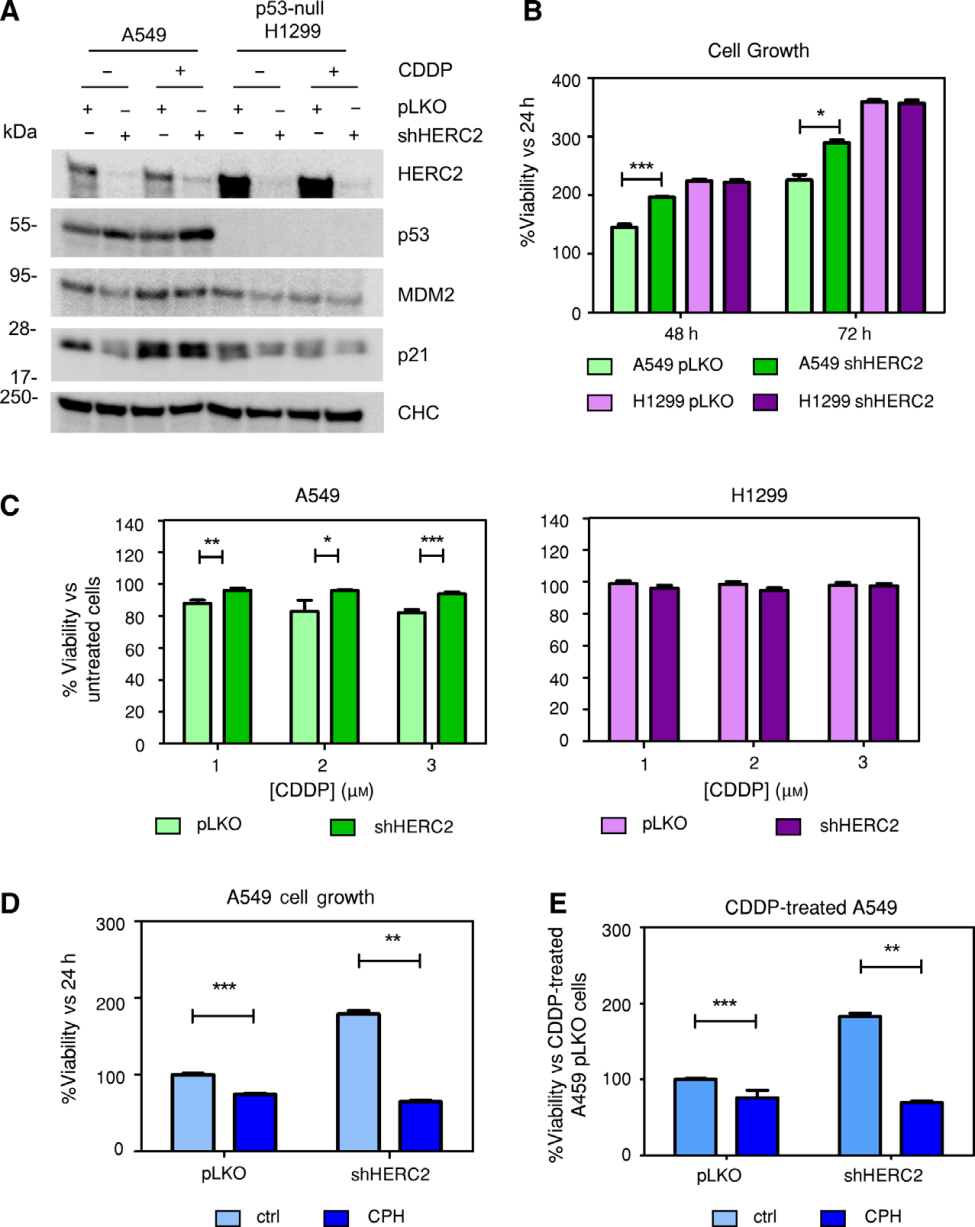
Stable HERC2 ablation confers a higher growth rate and reduced DNA damage sensitivity in *wt* p53 contexts. (A) p53‐*wt* A549 and p53‐null H1299 cells were infected with lentivirus carrying either HERC2‐directed shRNA (shHERC2) or empty pLKO vector (pLKO) as control. After selection with puromycin, cells were treated with 3 µm cisplatin (CDDP) for 48 h. Protein extracts were immunoblotted against the indicated proteins. Clathrin heavy chain (CHC) was used as loading control. (B) HERC2‐knockdown and control pLKO A549 and H1299 cells were seeded and checked for viability using the MTT method. The chart indicates viability rates compared to the 24‐h point. (C) Cells were seeded as in (B). 24 h after seeding, cells were treated with the indicated concentrations of cisplatin for 48 h and viability was assessed using the MTT method. The charts indicate survival rates compared to those of untreated cells. (D) A549 pLKO and shHERC2 were transfected with the F3 fragment of HERC2 (residues 2292–2923 containing the CPH domain), and viability was measured 72 h after transfection by MTT method. (E) A549 cells were transfected as in (D). Twenty‐four hours after transfection, cells were treated with 1.5 µm CDDP and incubated for additional 48 h prior MTT staining. Results are the mean of two independent experiments. Data were analyzed by Student’s *t*‐test. Error bars indicate SEM from three independent experiments.

As argued above and in other articles (Cubillos‐Rojas *et al.*, 2014), HERC2 is crucial for correct p53 transcriptional activity. Hence, we decided to investigate whether stable *HERC2* silencing would result in the impairment of p53 physiological functions such as cell proliferation regulation or cell death triggered by DNA damage. Non‐small‐cell lung cancer (NSCLC) A549 (*wt* p53) and H1299 (p53‐null) cell lines were infected with lentivirus carrying either an empty vector (pLKO) or shRNA against *HERC2* (shHERC2). To further investigate the effects on p53 functionalities, we took advantage of *cis*‐diamminedichloro platinum (II) (CDDP), also known as cisplatin, which is a widely used chemotherapeutic drug that induces DNA damage and apoptotic cell death in *wt* p53 contexts (Fennell *et al.*, 2016; García‐Cano *et al.*, 2015; Macciò and Madeddu, 2013). HERC2 expression was assessed by western blot following selection (Fig. 7A). In A549 shHERC2 cells, MDM2 and p21 levels are drastically reduced. This decrease is partially recovered upon cisplatin treatment. None of this is evident in p53‐null H1299 cells. *HERC2* silencing stimulated cell growth in A549 cells but had no significant effect in H1299 as measured by MTT (Fig. 7B). Similar results were obtained by crystal violet method (Fig. S2). Clonogenic assays also performed by crystal violet suggest that these phenotypes are maintained in long‐term colony formation cultures (Fig. S3). *HERC2*‐silenced A549 cells showed higher resistance against cisplatin treatment than their control counterparts. However, sensitivity toward cisplatin remained unchanged in H1299 when interfering *HERC2* versus no interference by MTT (Fig. 7C) with similar tendencies by crystal violet method (Fig. S4), thus confirming that HERC2 is necessary for the effect that p53 exerts on proliferation and cytotoxic response to chemotherapeutic drugs. To further demonstrate that these effects rely on the presence of HERC2 and p53 rather than in any other differences among cell lines, we carried out phenotype rescue experiments in which A549 cells (both pLKO and shHERC2) were transfected with the CPH‐containing F3 fragment of HERC2 (residues 2292–2923) used in Fig. 1C. CPH domain ectopic expression modestly reduces cell growth in HERC2‐intact A549 pLKO cells. However, this parameter is drastically diminished from HERC2‐lacking cells upon CPH transfection (Fig. 7D). Regarding response of A549 cells to cisplatin, CPH domain introduction could also rescue HERC2 knockdown cells. As it is evident in Fig. 7E, CPH transfection, which mildly sensitizes A549 pLKO cells, strongly reverts shHERC2 protection against cisplatin resembling once more the nonsilenced phenotype. These data confirm that HERC2 is necessary for complete functionalities of p53 in cell contexts such as cell growth and response to DNA‐damaging drug treatment. (Raw data from this set of experiments are available in Fig. S5).

## 4 Discussion

The ubiquitin ligase HERC2 forms a complex with the NEURL4 protein that stabilizes NEURL4 (Al‐Hakim *et al.*, 2012; Cubillos‐Rojas *et al.*, 2017; Galligan *et al.*, 2015). This complex interacts with the p53 tumor suppressor protein and regulates its transcriptional activity by regulating its oligomerization (Cubillos‐Rojas *et al.*, 2014, 2017). In the process of activating the transcription of genes regulated by p53, oligomerization of p53 precedes its acetylation (Itahana *et al.*, 2009), the acetylation being indispensable for its transcriptional activity (Tang *et al.*, 2008). In this model, HERC2 and NEURL4 function as essential factors for the oligomerization of p53. In nonstressed cells, p53 is normally kept under control by the ubiquitin ligase MDM2. p53 and MDM2 form a negative feedback loop in which p53 activates the transcription of *MDM2*, and MDM2 ubiquitylates p53 which inhibits its transcriptional activity, facilitates its cytoplasmatic localization, and promotes its degradation (Karni‐Schmidt *et al.*, 2016; Manfredi, 2010). Here, we demonstrate that under nonstress conditions, the ubiquitin ligase MDM2 is part of the complex formed by HERC2, NEURL4, and p53. The interaction of MDM2 with this complex is mediated by p53 since the interaction was not observed in p53‐null H1299 cells and transfection of *wt* p53 in these cells recovered the interaction. The transfection of mutant forms of p53 that cannot form tetramers demonstrated that p53 must be in a tetrameric form for MDM2 to interact and be part of the complex with HERC2 and NEURL4. Previous reports showing that the p53 tetramerization domain is required for efficient ubiquitylation by MDM2 (Maki, 1999) and that p53 tetramers can be ubiquitylated (Brooks *et al.*, 2007), are consistent with these observations. Under stress conditions caused by bleomycin‐induced DNA damage, p53 is phosphorylated and acetylated, remaining bound to the complex while MDM2 is dissociated. These data are consistent with previous studies showing that kinases activated by DNA damage such as ATM or ATR phosphorylate MDM2 and p53, inhibiting MDM2 ability to polyubiquitylate p53 (reviewed by Cheng and Chen, 2010). Furthermore, DNA damage kinases induce MDM2 self‐degradation (Stommel and Wahl, 2004). Since ATM, ATR, and DNA‐PK interact with HERC2 (Bekker‐Jensen *et al.*, 2010), it is possible that the binding of these kinases to the HERC2‐p53‐MDM2 complex is necessary to phosphorylate MDM2 and p53. The phosphorylation of the carboxyl end of HERC2 by these kinases (Bekker‐Jensen *et al.*, 2010) could also be involved in the MDM2 release mechanism of the HERC2‐p53‐MDM2 complex after DNA damage. On the other hand, p53 acetylation does not occur on p53 mutants that are incapable of forming tetramers because acetyltransferases cannot interact with them (Itahana *et al.*, 2009). Finally, consistent with previous reports (Cubillos‐Rojas *et al.*, 2014; Kawaguchi *et al.*, 2006; Stommel *et al.*, 1999; Weinberg *et al.*, 2004), p53 exists largely in the dimeric form in nonstressed U2OS cells. Upon stress signaling caused by DNA damage by bleomycin, a fraction of p53 shifts to the tetramer form in a phosphorylated and acetylated state and binds more efficiently to DNA and activates p53 target genes such as *p21* or *MDM2*.

**Figure 8:**
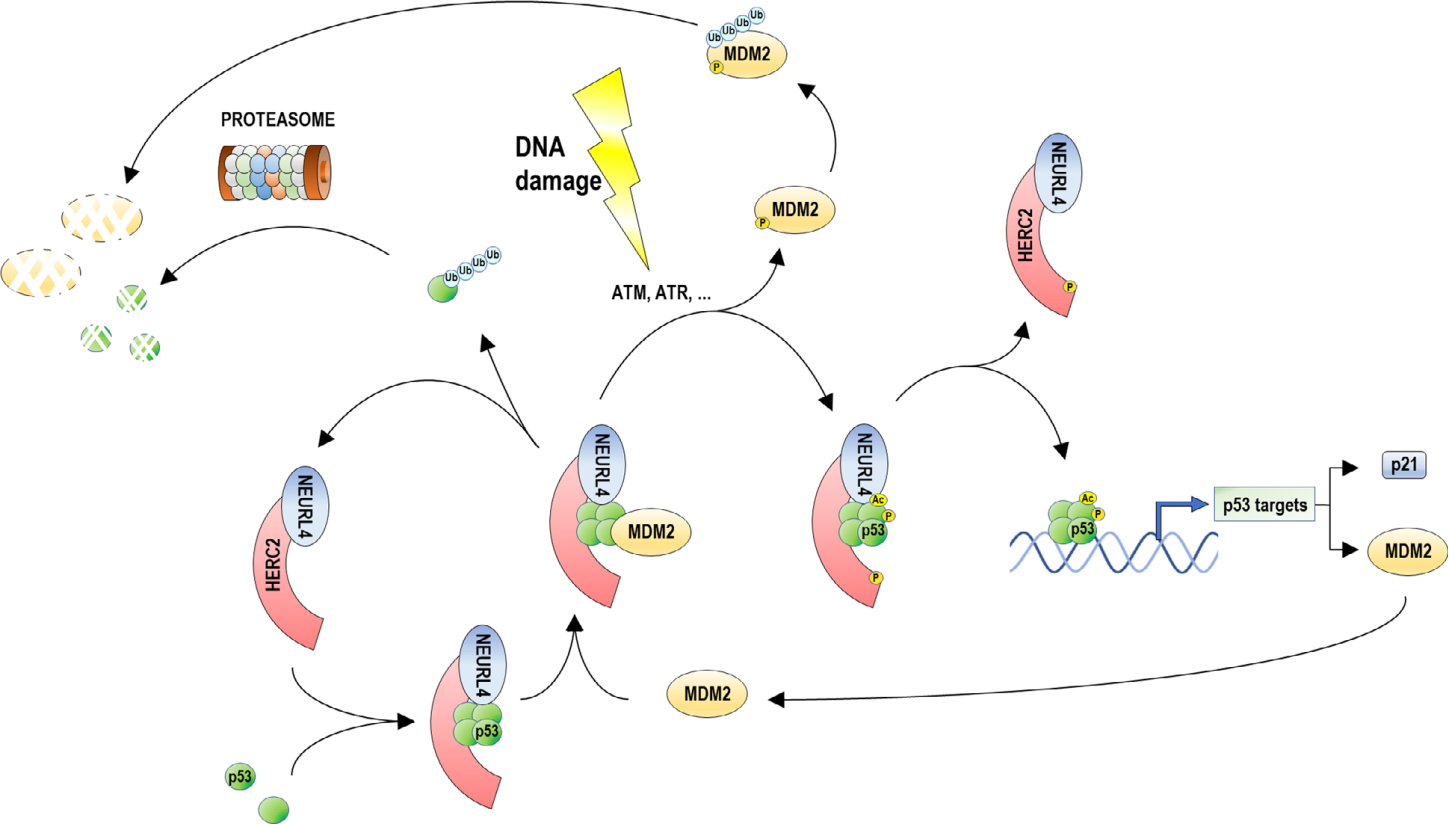
Working model of the role of HERC2 in p53‐MDM2 loop regulation. The HERC2‐NEURL4 complex binds oligomerized p53 through the CPH domain of HERC2. The p53‐HERC2‐NEURL4 complex binds MDM2 and, in the basal state, MDM2 polyubiquitylates p53, thus marking it for proteasomal degradation. Upon DNA damage (such as bleomycin treatment), DNA damage‐sensing kinases phosphorylate MDM2 and HERC2 thus detaching MDM2 from the complex. Phosphorylated MDM2 is unstable, is autopolyubiquitylated, and later is degraded by proteasome as well. This situation also induces activation of p53, in terms of phosphorylation and acetylation, and, later, its translocation to the promoters of its targeted genes such as *p21* or *MDM2* itself the protein product of which can bind p53 and begin the regulatory loop again.

Together, our findings reveal the importance of HERC2 in regulating the p53‐MDM2 loop and suggest a model (Fig. 8) whereby HERC2 functions at least at three levels. First, HERC2 together with NEURL4 is necessary for p53 to tetramerize, forming a HERC2‐NEURL4‐p53 complex. At this stage, HERC2 would function as a stimulator of oligomerization through its CPH domain. Second, the existence of the HERC2‐NEURL4‐p53 complex allows the interaction of MDM2 with tetramerized p53, which enables MDM2‐dependent p53 ubiquitylation that results in proteasomal degradation. In response to DNA damage, this complex would also allow MDM2, p53, and HERC2 phosphorylation, MDM2 release, and p53 acetylation. At this stage, HERC2 would function as a scaffolding factor that allows the recruitment of all these proteins, a previously suggested function for the interaction between HERC2 and NEURL4 (Galligan *et al.*, 2015). Third, the location in the nucleus of the HERC2‐p53 complex (Cubillos‐Rojas *et al.*, 2014) allows acetylated, phosphorylated, and tetramerized p53 to interact with the p53‐binding sequences in the promoters of its target genes such as *p21* or *MDM2*. This interaction releases p53 from the complex with HERC2 and activates the transcription of the target genes that, in the case of *MDM2*, produces a negative feedback loop regulation of p53. At this stage, HERC2 would participate in the transcription activation process to facilitate the binding of p53 to its promoters.

Ubiquitin E3 ligases can be classified according to their ligase domains into three main types: RING, RING‐between‐RING (RBR), and HECT (Buetow and Huang, 2016). HERC2 belongs to the HECT family and MDM2 is a member of the RING family. Here, we show that the ubiquitin ligases HERC2 and MDM2 form a complex with p53. HERC2 functions as a positive modulator stimulating p53 oligomerization whereas MDM2 functions as a negative modulator regulating p53 ubiquitylation. p53 can be a substrate of several ubiquitin E3 ligases, but it seems that MDM2 is the main ubiquitin E3 ligase for p53 (Haupt *et al.*, 1997; Kubbutat *et al.*, 1997; Michael and Oren, 2003). MDM2 ubiquitylates p53 thus labeling it for nuclear‐cytoplasmatic shuttle (monoubiquitylation) or for proteasome‐dependent degradation (polyubiquitylation) (Li, 2003). Autopolyubiquitylation and later proteasome‐dependent degradation have been described as the main pathway of MDM2 degradation (Fang *et al.*, 2000; Honda and Yasuda, 2000). Because MDM2 knockdown does not regulate HERC2 levels, it is likely that MDM2 does not ubiquitylate HERC2 for proteasome‐dependent degradation. HERC2 depletion experiments show that p53 and MDM2 protein levels do not increase, suggesting that HERC2 does not ubiquitylate these proteins for proteasome‐dependent degradation. Genetic studies have confirmed the physiological importance of these genes. Thus, while *TP53* knockout mice are viable despite being prone to developing tumors (Donehower *et al.*, 1992; Jacks *et al.*, 1994), *MDM2* or *HERC2* knockout mice are lethal during embryonic phase (Cubillos‐Rojas *et al.*, 2016; Jones *et al.*, 1995; Montes de Oca Luna *et al.*, 1995). *TP53* knockout mice can rescue *MDM2* knockout mice (Jones *et al.*, 1995; Montes de Oca Luna *et al.*, 1995) but they cannot rescue *HERC2* knockout mice (Cubillos‐Rojas *et al.*, 2016). These results suggest that an increment in p53 levels during the embryonic phase is the cause for the unviability of *MDM2* knockout animals. In the case of *HERC2* knockout mice, these data imply that HERC2 has an essential role during development and that this function is independent of its regulation of p53 activity. HERC2 ubiquitylation substrates could be involved in the essential function of HERC2 during development. In this sense, proteins involved in DNA repair mechanisms (such as XPA, Kang *et al.*, 2010 and BRCA1, Wu *et al.*, 2010) and in iron homeostasis (such as FBXL5, Moroishi *et al.*, 2014) are targeted by HERC2 for proteasome‐dependent degradation. BRCA1 or FBXL5 deficiency results in early embryonic lethality in the same way as HERC2 deficiency (Gowen *et al.*, 1996; Liu *et al.*, 1996; Moroishi *et al.*, 2011). Although these HERC2 substrates are expected to be increased in HERC2 knockout mice, we cannot discard a lethality by a dysregulation of their cellular functions by overexpression.

p53 functions as a tumor suppressor that protects cells from malignant transformation, and its inactivation is associated with tumorigenesis. The importance of p53 in human cancer is evident given that the *TP53* gene is mutated in about half of all sporadic cancers and in patients with Li‐Fraumeni syndrome, who are cancer prone (Li and Fraumeni, 1969) and harbor germline mutations in the *TP53* gene (Freed‐Pastor and Prives, 2012; Manfredi, 2010). Analysis of p53 mutations in patients with Li‐Fraumeni syndrome revealed that the mutation frequency relative to the length of the DNA binding domain and in the oligomerization domain is almost the same (Kamada *et al.*, 2011, 2016). Because mutations in Li‐Fraumeni syndrome occur within the oligomerization domain with considerable frequency, it has been proposed that transcriptional defects and deregulated MDM2 circuitry are likely contributors to this pathology (Katz *et al.*, 2018). Our data suggest that HERC2 could also protect cells from malignant transformation. In this context, mutations in HERC2 have been detected in T‐cell prolymphocytic leukemia (Johansson *et al.*, 2018) and in gastric and colorectal carcinomas with microsatellite instability (Yoo *et al.*, 2011). Moreover, the *HERC2* locus has been associated with cutaneous melanoma (Amos *et al.*, 2011) and uveal melanoma (Ferguson *et al.*, 2016). In tumors with wt p53, an attractive approach is to reactivate p53. Nutlin‐3 can promote this reactivation by blocking the MDM2‐p53 interaction (Vassilev, 2004). Several Nutlin‐3 analogs are in clinical trials for treatment of human cancers (Burgess *et al.*, 2016; Zhao *et al.*, 2015). Another way in which to reactivate p53 is by stimulating its oligomerization. Drugs causing nongenotoxic activation of p53 oligomerization may be potential candidates for cancer therapy. In this context, induction of HERC2 activity leading to higher p53 oligomerization may be a potential target for cancer therapy.

## 5 Conclusions

MDM2 ubiquitin E3 ligase forms a complex along with HERC2 and NEURL4 necessarily through oligomerized p53. *HERC2* knockdown results in reduced *MDM2* promoter activation and, hence, diminished *MDM2* mRNA expression in *wt* p53 contexts. Upon bleomycin‐induced DNA damage, first MDM2 is released of the oligomeric p53/HERC2/NEURL4 complex, and then, p53 response elements‐containing promoters compete with the HERC2‐NEURL4 tandem for active p53 binding. Functional HERC2 is required for the maintenance of p53 activity in terms of cell growth control and response to cisplatin‐induced cell death.

## Conflict of interest

The authors declare no conflict of interest.

## Author contributions

JG‐C, SS‐T, and JLR conceived and designed the project. JG‐C, SS‐T, JS‐G, and AF performed all the experiments and analyzed the data. JLR and JG‐C wrote the manuscript. FVi, RB, FVe, and JLR worked on the original idea and helped edit the paper and obtain funding. All authors discussed the results and commented on the manuscript.

## Supporting information


**Fig. S1.** Bleomycin treatment activates DNA damage‐response pathway.

**Fig. S2.** Crystal violet staining yields similar results to those of MTT in cell proliferation assay.

**Fig. S3.** Clonogenic assay confirms cell growth promotion upon *HERC2* gene stable silencing.

**Fig. S4.** Crystal violet staining yields similar results to those of MTT in CDDP treatment assay.

**Fig. S5.** Raw data from Figure 7.
