# Supplementary figures and images for: Regulation of the MDM2‐p53 pathway by the ubiquitin ligase HERC2
Source: Mol Oncol. 2019 Nov 15;14(1):69–86. doi: 10.1002/1878-0261.12592 (PMC6944118; doi:10.1002/1878-0261.12592)

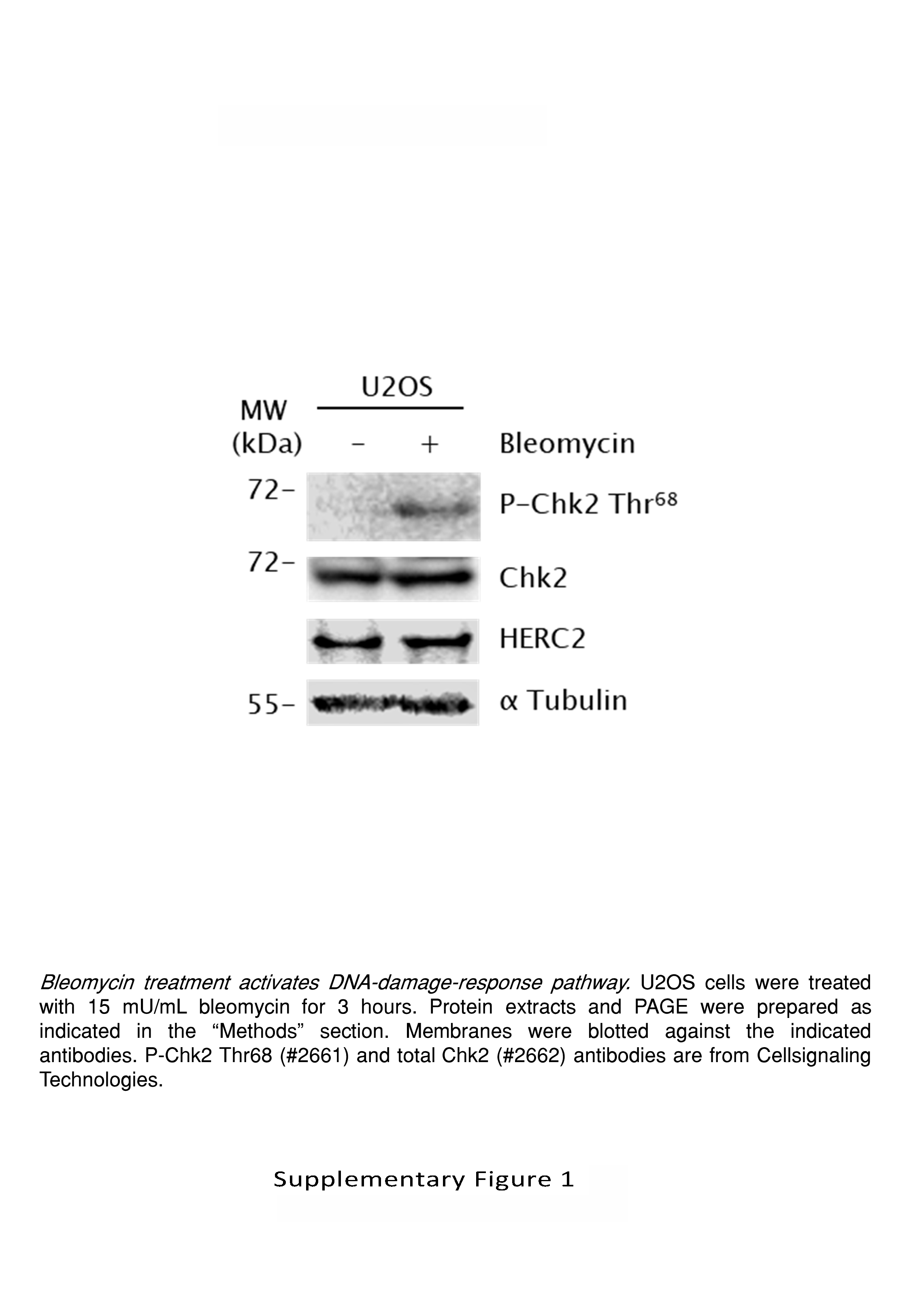

Supplement: Supplementary file 1 — Fig. S1. Bleomycin treatment activates DNA damage‐response pathway. [file MOL2-14-69-s001.TIF]

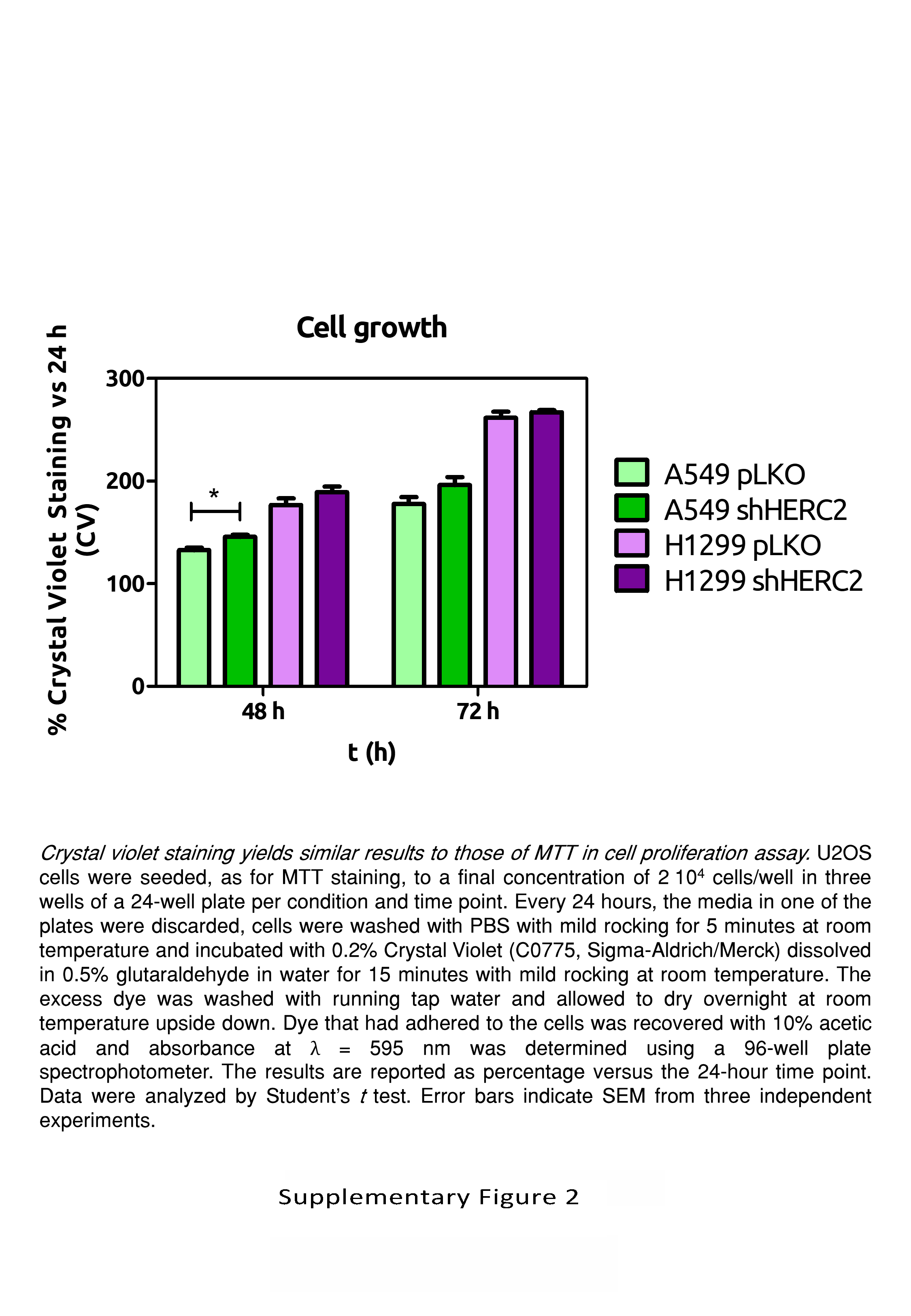

Supplement: Supplementary file 2 — Fig. S2. Crystal violet staining yields similar results to those of MTT in cell proliferation assay. [file MOL2-14-69-s002.TIF]

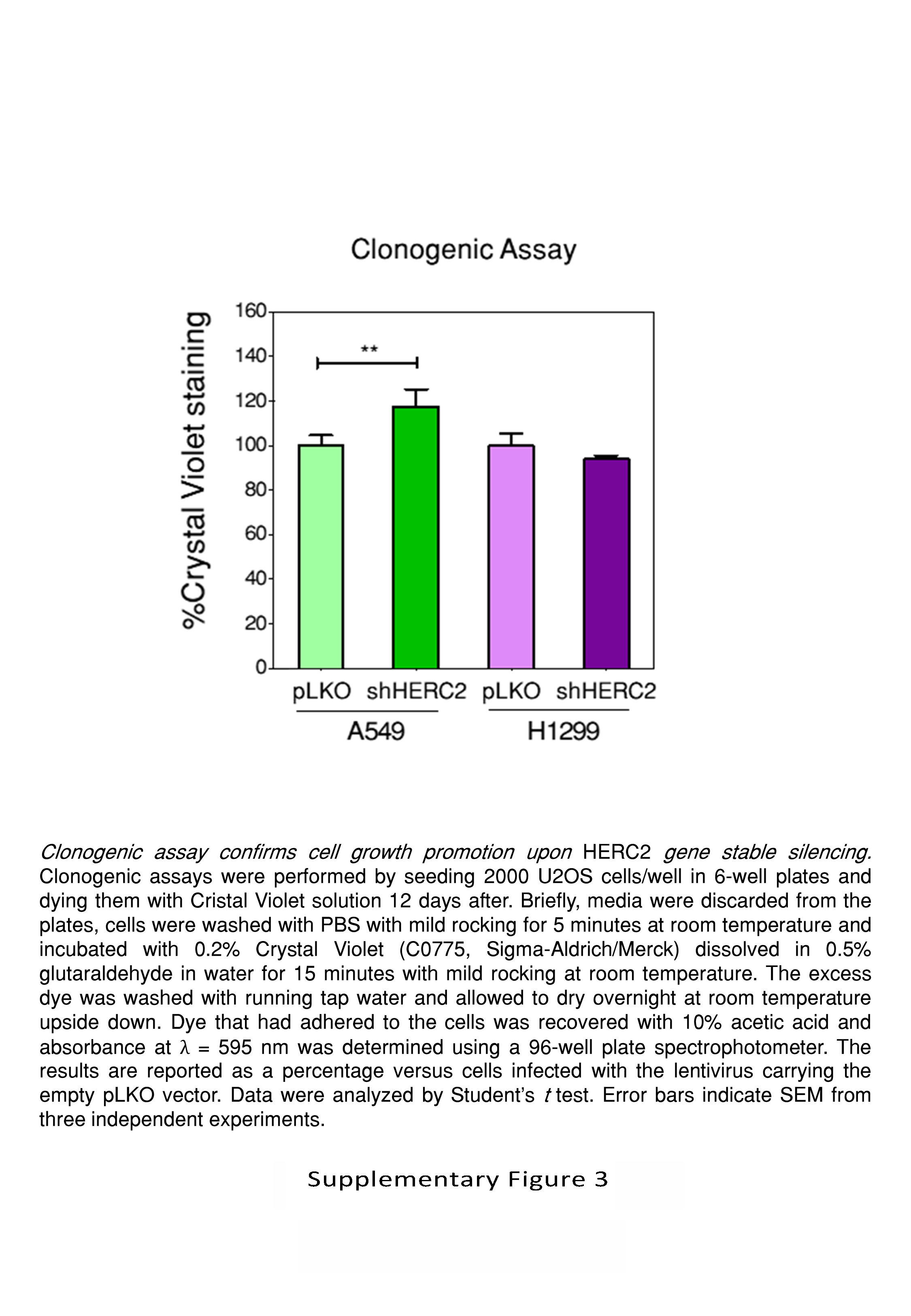

Supplement: Supplementary file 3 — Fig. S3. Clonogenic assay confirms cell growth promotion upon HERC2 gene stable silencing. [file MOL2-14-69-s003.TIF]

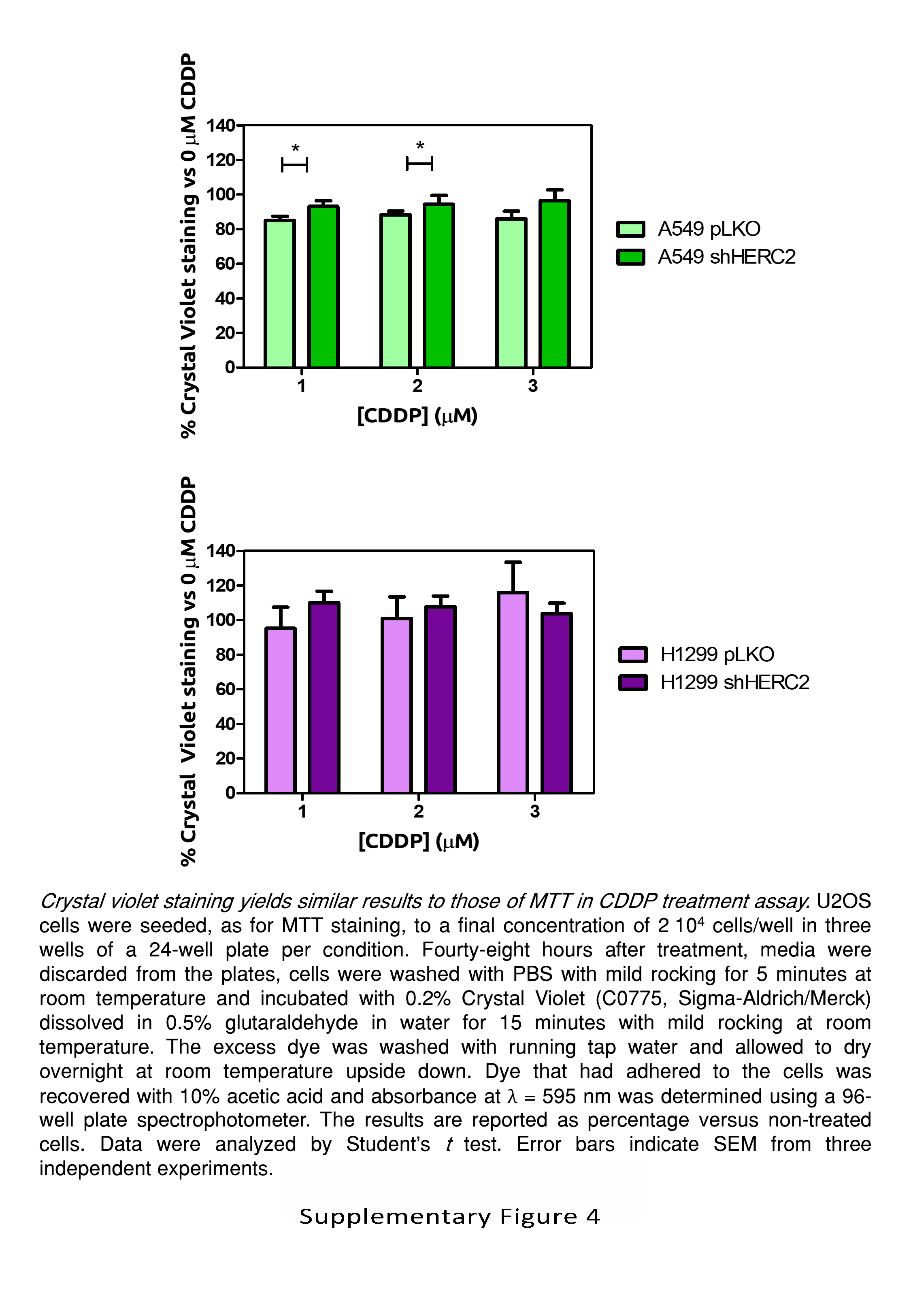

Supplement: Supplementary file 4 — Fig. S4. Crystal violet staining yields similar results to those of MTT in CDDP treatment assay. [file MOL2-14-69-s004.TIF]

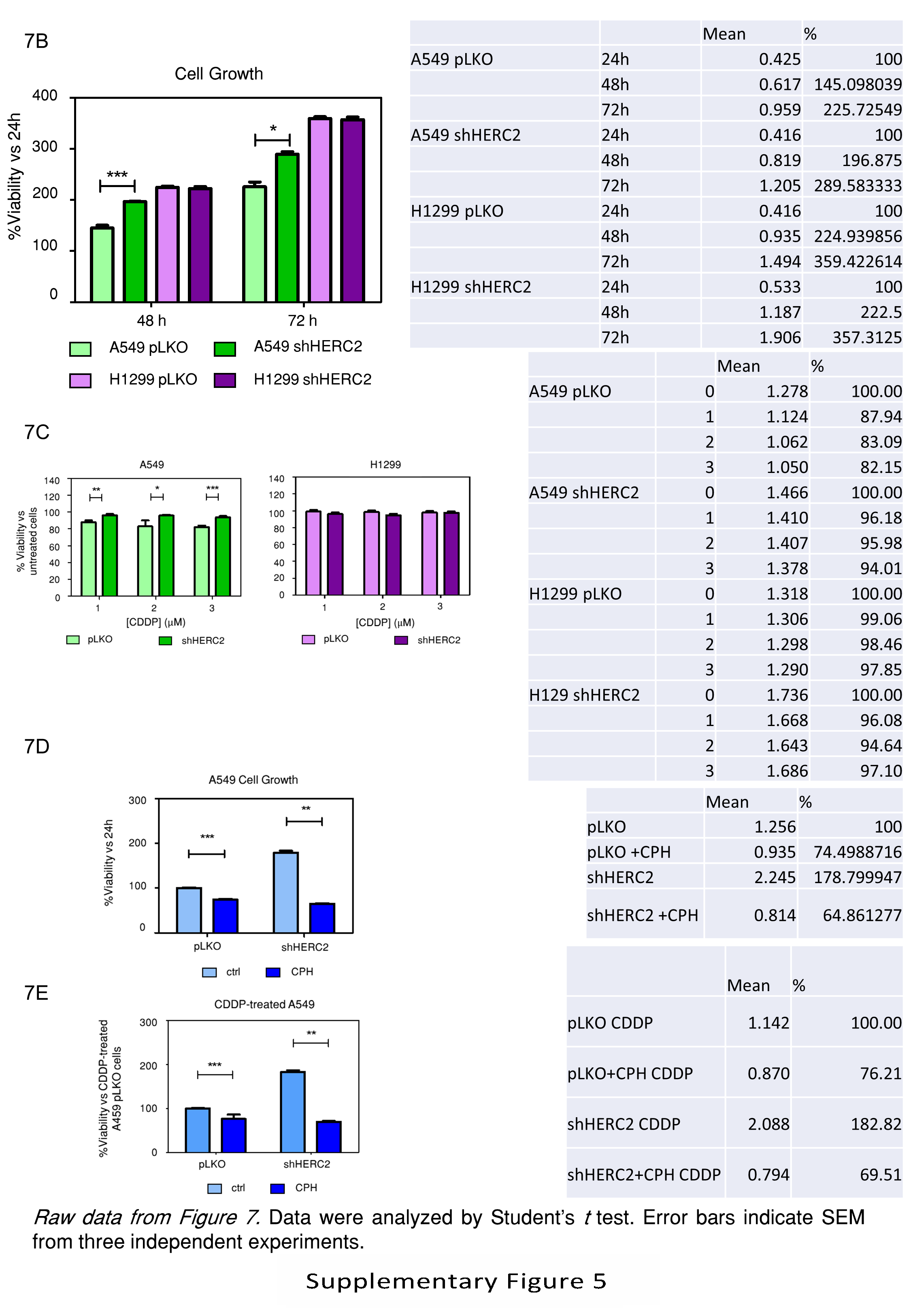

Supplement: Supplementary file 5 — Fig. S5. Raw data from Figure 7. [file MOL2-14-69-s005.TIF]
